# Supplementary material for: A Genetically Encoded Dark-to-Bright Biosensor for Visualisation of Granzyme-Mediated Cytotoxicity
Source: Int J Mol Sci. 2023 Sep 2;24(17):13589. doi: 10.3390/ijms241713589 (PMC10487497; doi:10.3390/ijms241713589)
Supplement: Supplementary file 1 [file ijms-24-13589-s001.zip › ijms-2584525-supplementary.pdf]

International Journal of *Molecular Sciences*

MDPI

Supplementary Materials

A Genetically Encoded Dark-to-Bright Biosensor for Visualisation of Granzyme-Mediated Cytotoxicity

Christopher Bednar, Sabrina Kübel, Arne Cordsmeier, Brigitte Scholz, Hanna Menschikowski and Armin Ensser *

Institute of Clinical and Molecular Virology, University Hospital Erlangen, Friedrich-Alexander-Universität Erlangen-Nürnberg, 91054 Erlangen, Germany; christopher.bednar@uk-erlangen.de (C.B.); sabrina.kuebel@uk-erlangen.de (S.K.); arne.cordsmeier@uk-erlangen.de (A.C.); brigitte.scholz@uk-erlangen.de (B.S.); hanna.menschikowski@uk-erlangen.de (H.M.)

* Correspondence: armin.ensser@fau.de

Table S1. Plasmids. The following table provides information to all plasmids which were used in this study.

Plasmid name	Origin
pCDNA3.1(-)	Thermo Fisher Invitrogen™ V79520
pGZMB	This work
pGZMBΔGE	This work
pLCTP-iCasp8FT	This work
pLCTP-iGZMBΔGE	This work
pLenti-CMV-rtTA3-Blast	Addgene #26429
pLenti-CMVtight-Puro-DEST	Addgene #26439
pLV-EF1α-IRES-Hygro	Addgene #85134
pLV-EF1α-IRES-Puro	Addgene #85132
pLV-EIH-CRSTAL	This work
pLV-EIP-CD19	This work
pMD2.G	Prof. Didier Trono, École Polytechnique Fédérale de Lausanne
pMSCV-CD19FBBz	This work
pMSCVpuro-DEST	Addgene #119745
psPAX2	Prof. Didier Trono, École Polytechnique Fédérale de Lausanne

Table S2. Oligonucleotide primers. The following table provides information to all oligonucleotides which were used in this study.

Primer name	Sequence (5′ → 3′)
ATG-Kozak-Not-as	CATGGTGGCGGCGGCCGCCACTGTGC
BamHIGZMB-fwd	AAAGGATCCGCCATGCAACCAATCCTGCTTCTGC
GZMBdGE-fwd	TGCTGCCCAGGGCAGATGCAATCATCGGGGGACATGAGGC
GZMBdGE-rev	GCCTCATGTCCCCCGATGATTGCATCTGCCCTGGGCAGCA
GZMBFlagAcc65I-rev	AAAGGTACCTACTTGTCGTCGTCGTCCTTGTAGTCG-TAGCGTTTCATGGTTTTCTTTATC
pLV-CD19-fwd	CAGTGGCGGCCGCCGCCACCATGCCAC-CTCCTCGCCTCCTCTTCTTC
pLV-CD19-rev	GGCTGATCAGCGGGTTTAAACTCACCTGGTGCTCCAGGTGCCCAT
Stop-Pme-LV-IRES	TGAGTTTAAACCCGCTGATCAGCCTCTAGCAAC-GGTTTCCCTCTAGCGG

Table S3. Genetic constructs. The following table provides information to all novel DNA constructs which were designed and generated in this study. The amino acid sequence is depicted in one-letter-code.

**Construct name**	**Amino acid sequence**
CD19FBBz	MASPLTRFLSLNLLLLGESIILGSGEADIQLTQSPASLAVSLGQRATISCKASQSVDY DGDSYLNWYQQIPGQPPKLLIYDASNLVSGIPPRFSGSGSGTDFTLNIHPVEKVDA ATYHCQQSTEDPWTFGGGTKLEIKGGGGSGGGGSGGGGSQVQLQQSGAELVRPG SSVKISCKASGYAFSSYWMNWVKQRPGQGLEWIGQIWPGDGDTNYNGKFKGKA TLTADESSSTAYMQLSSLASEDSAVYFCARRETTTVGRYYYAMDYWGQGTTVTVS SAAADYKDDDDKTTTPAPRPPTPAPTIASQPLSLRPEACRPAAGGAVHTRGLDFA CDIYIWAPLAGTCGVLLLSLVITLYCKRGRKKLLYIFKQPFMRPVQTTQEEDGCSCR FPEEEEGGCELRVKFSRSADAPAYKQGQNQLYNELNLGRREEYDVLDKRRGRDPE MGGKPRRKNPQEGLYNELQKDKMAEAYSEIGMKGERRRGKGHDGLYQGLSTAT KDTYDALHMQALPPR
CRSTAL	MIKIATRKYLGKQNVYDIGVERDHNFALKNGFIASNCFNKTIISTFKWSYTTVNGK RYRSTARTTYTFAKPMAANYLKNQPMYVFRKTELKHSMTELNFKEWQKAFTDIE PDSGEDNMASLPATHELHIFGSINGVDFDMVGQGTGNPNDGYEELNLKSTKGDL QFSPWILVPHIGYGFHQYLPYPDGMSPFQAAMVDGSGYQVHRTMQFEDGASLTV NYRYTYEGSHIKGEAQVMGTGFPADGPVMTNTLTAADWCMSKKTYPNDAEYCL SYETEILTVEYGLLPIGKIVEKRIECTVYSVDNNGNIYTQPVAQWHDRGEQEVFEYC LEDGSLIRATKDHKFMTVDGQMLPIDEIFERELDLMRVDNLPNSHGFPPEVEEQD DGTLPMSCAQESGMDRHPAACASARINV
GZMBΔGE	MQPILLLLAFLLLPRADAIIGGHEAKPHSRPYMAYLMIWDQKSLKRCGGFLIRDDF VLTAAHCWGSSINVTLGAHNIKEQEPTQQFIPVKRAIPHPAYNPKNFSNDIMLLQ LERKAKRTRAVQPLRLPSNKAQVKPGQTCSVAGWGQTAPLGKHSHTLQEVKMT VQEDRKCESDLRHYYDSTIELCVGDPEIKKTSFKGDSGGPLVCNKVAQGIVSYGRN NGMPPRACTKVSSFVHWIKKTMKRYDYKDDDDK
iCasp8FT	MDYKDDDDKSESQTLDKVYQMKSKPRGYCLIINNHNFAKAREKVPKLHSIRDRN GTHLDAGALTTTFEELHFEIKPHDDCTVEQIYEILKIYQLMDHSNMDCFICCILSHG DKGIIYGTDGQEAPIYELTSQFTGLKCPSLAGKPKVFFIQACQGDNYQKGIPVETDR RKRGSGEGRGSLLTCGDVEENPGPLSSPQTRYIPDEADFLLGMATVNNCVSYRNP AEGTWYIQSLCQSLRERCPRGDDILTILTEVNYEVSNKDDKKNMGKQMPQTFTL RKKLVFPSD

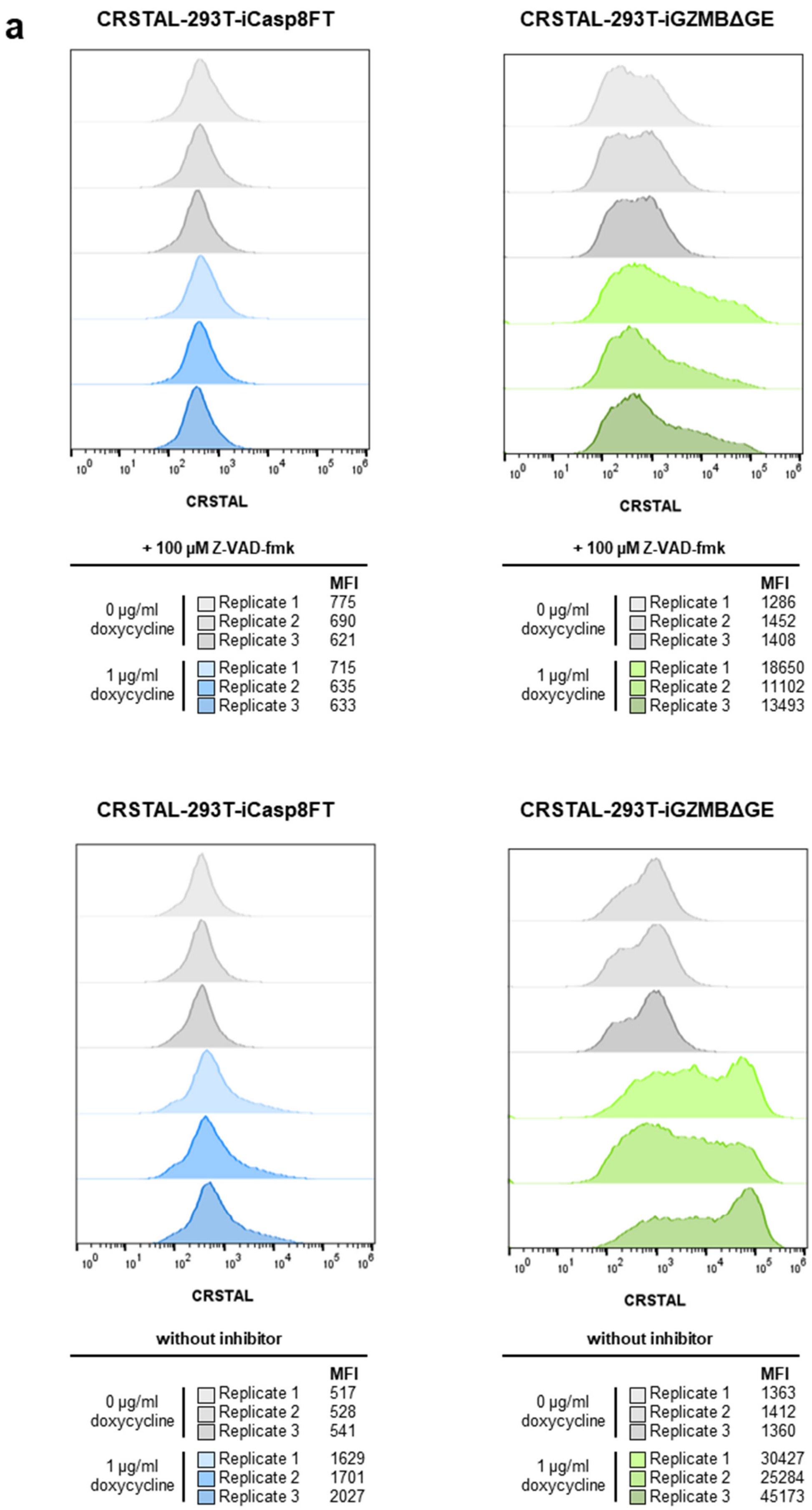
a
CRSTAL-293T-iCasp8FT
CRSTAL-293T-iGZMBΔGE
CRSTAL
+ 100 µM Z-VAD-fmk
MFI
0 µg/ml doxycycline
Replicate 1 775
Replicate 2 690
Replicate 3 621
1 µg/ml doxycycline
Replicate 1 715
Replicate 2 635
Replicate 3 633
+ 100 µM Z-VAD-fmk
MFI
0 µg/ml doxycycline
Replicate 1 1286
Replicate 2 1452
Replicate 3 1408
1 µg/ml doxycycline
Replicate 1 18650
Replicate 2 11102
Replicate 3 13493
CRSTAL-293T-iCasp8FT
CRSTAL-293T-iGZMBΔGE
CRSTAL
without inhibitor
MFI
0 µg/ml doxycycline
Replicate 1 517
Replicate 2 528
Replicate 3 541
1 µg/ml doxycycline
Replicate 1 1629
Replicate 2 1701
Replicate 3 2027
without inhibitor
MFI
0 µg/ml doxycycline
Replicate 1 1363
Replicate 2 1412
Replicate 3 1360
1 µg/ml doxycycline
Replicate 1 30427
Replicate 2 25284
Replicate 3 45173

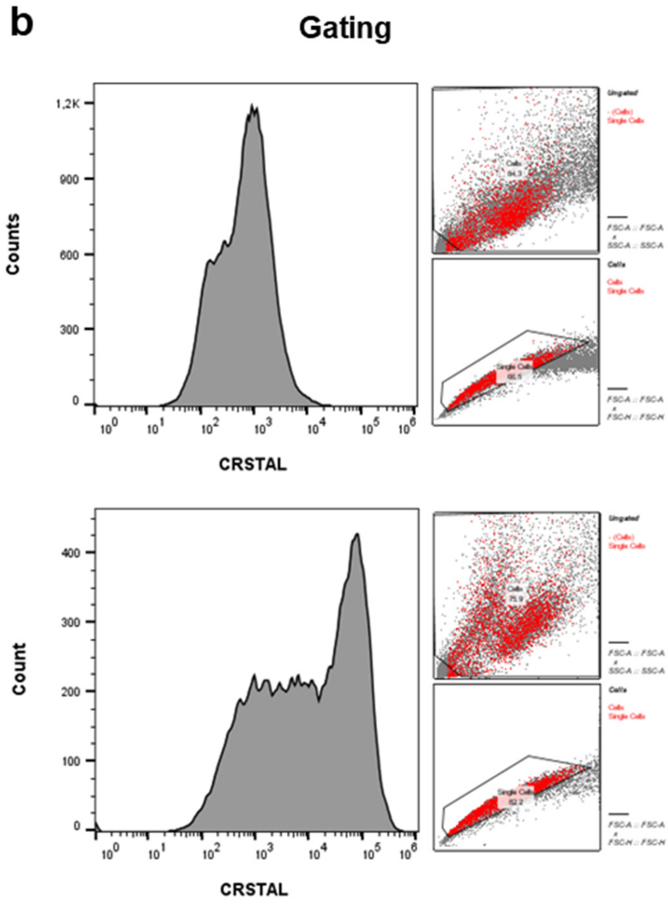

Figure S1: Detailed flow cytometry data related to Figure 5. Expression of active caspase-8 or GZMB was induced in CRSTAL-293T-iCasp8FT or CRSTAL-293T-iGZMBΔGE via treatment with 1 µg/ml doxycycline in presence or absence of 100 µM Z-VAD-fmk. Cells were analysed 48 h post induction via flow cytometry. (**a**) Histograms showing the CRSTAL fluorescence signal in treated CRSTAL-293T-iCasp8FT or CRSTAL-293T-iGZMBΔGE cells. MFI values are listed in the tables below. (**b**) Gating strategy applied in flow cytometric analyses.

z

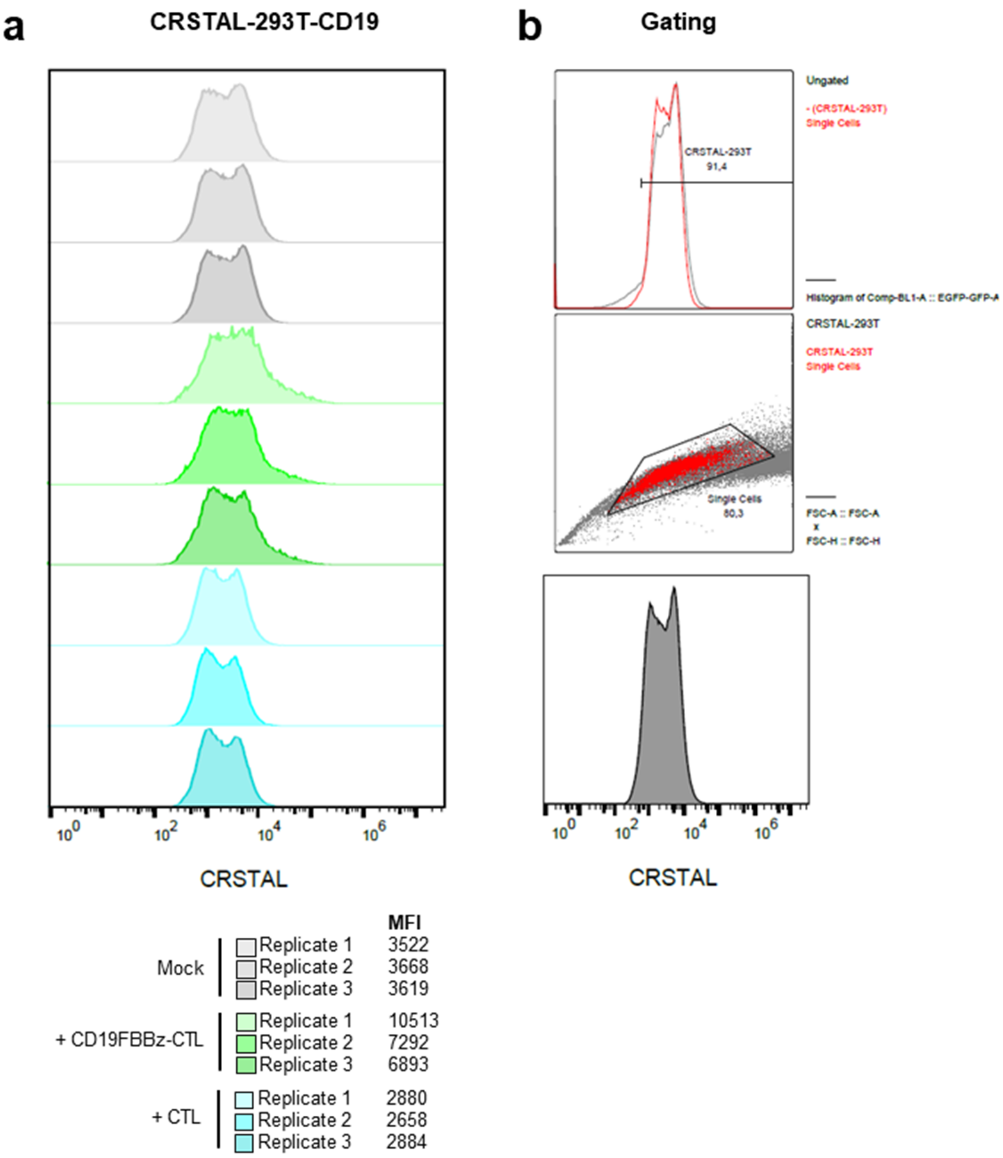

Figure S2: Detailed flow cytometry data related to Figure 6. CRSTAL-293T-CD19 cells were seeded and co-incubated with CD19FBBz-CTL or CAR-negative CTL at an effector to target ratio of 5:1 for 48 h. Cells were stained and analysed via flow cytometry. (**a**) Histograms showing the CRSTAL fluorescence signal in CRSTAL-293T-CD19 co-incubated with CAR-T cells, non-CAR-T cells or no T cells. MFI values are listed in the table below. (**b**) Gating strategy applied in flow cytometric analyses.
